# Supplementary figures and images for: Effect of umbilical cord blood stem cell transplantation on restenosis after endovascular interventional therapy for diabetic hindlimb vascular disease
Source: PLoS One. 2021 Aug 11;16(8):e0255162. doi: 10.1371/journal.pone.0255162 (PMC8357084; doi:10.1371/journal.pone.0255162)

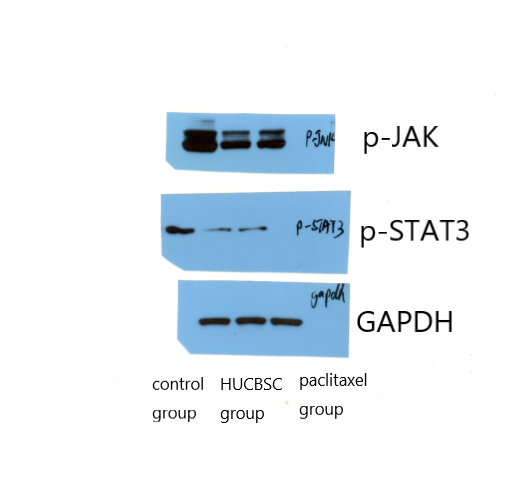

Supplement: S1 Fig — (TIF) [file pone.0255162.s001.tif]
