# Supplementary material for: Effect of umbilical cord blood stem cell transplantation on restenosis after endovascular interventional therapy for diabetic hindlimb vascular disease
Source: PLoS One. 2021 Aug 11;16(8):e0255162. doi: 10.1371/journal.pone.0255162 (PMC8357084; doi:10.1371/journal.pone.0255162)
Supplement: Attachment — Submitted filename: response to review comments.docx [file pone.0255162.s002.docx]

Your manuscript files have been checked in-house but before we can proceed we need you to address the following issues:

1) It appears that your ORCiDiD has not been validated in your Editorial Manager account and we are unable to proceed until that step is complete.

To validate your ORCiDiD in Editorial Manager, please follow the steps below:

1. In your Editorial Manager account, please go to ‘Update my Information’ (in the upper left-hand corner of the main menu), and click on the Fetch/Validate link next to the ORCiD field.

2. This link will take you to the ORCiD site and allow you to create a new iD or authenticate a pre-existing iD in Editorial Manager.

For additional instructions, please watch the following video for a step-by-step demonstration: https://www.youtube.com/watch?v=_xcclfuvtxQ

**Reply**: Thanks for your comments.

2) Thank you for including your ethics statement on the online submission form: " The study protocol was approved by the Ethics Committee of the Fourth Hospital of Hebei Medical University. "

To help ensure that the wording of your manuscript is suitable for publication, would you please also add this statement at the beginning of the Methods section of your manuscript file.

**Reply**: Thanks for your comments. It has been added in the Methods section as follows:

The study protocol was approved by the Ethics Committee of the Fourth Hospital of Hebei Medical University. (Page 6, Line 150-151)

3) To comply with PLOS ONE submissions requirements, please provide the method of euthanasia in the Methods section of your manuscript.

**Reply**: Thanks for your comments. The method of euthanasia has been added in the Methods section. See as follows: The rabbits were euthanized by ear intravenous injection of pentobarbital sodium (100mg/kg) at the end of the experiment. (Page 6, Line 173-174)

4) Thank you for stating the following financial disclosure:

"no funding receive."

At this time, please address the following queries:

Please clarify the sources of funding (financial or material support) for your study. List the grants or organizations that supported your study, including funding received from your institution.

State what role the funders took in the study. If the funders had no role in your study, please state: “The funders had no role in study design, data collection and analysis, decision to publish, or preparation of the manuscript.”

If any authors received a salary from any of your funders, please state which authors and which funders.

If you did not receive any funding for this study, please state: “The authors received no specific funding for this work.”

Please include your amended statements within your cover letter; we will change the online submission form on your behalf.

**Reply**: Thanks for your comments.The authors received no specific funding for this work.

5) In your Data Availability statement, you have not specified where the minimal data set underlying the results described in your manuscript can be found. PLOS defines a study's minimal data set as the underlying data used to reach the conclusions drawn in the manuscript and any additional data required to replicate the reported study findings in their entirety. All PLOS journals require that the minimal data set be made fully available. For more information about our data policy, please see http://journals.plos.org/plosone/s/data-availability.

Upon re-submitting your revised manuscript, please upload your study’s minimal underlying data set as either Supporting Information files or to a stable, public repository and include the relevant URLs, DOIs, or accession numbers within your revised cover letter. For a list of acceptable repositories, please see http://journals.plos.org/plosone/s/data-availability#loc-recommended-repositories. Any potentially identifying patient information must be fully anonymized.

Important: If there are ethical or legal restrictions to sharing your data publicly, please explain these restrictions in detail. Please see our guidelines for more information on what we consider unacceptable restrictions to publicly sharing data: http://journals.plos.org/plosone/s/data-availability#loc-unacceptable-data-access-restrictions. Note that it is not acceptable for the authors to be the sole named individuals responsible for ensuring data access.

We will update your Data Availability statement to reflect the information you provide in your cover letter.

**Reply**: Thanks for your comments. The datasets generated and analyzed during the current study are available from the corresponding author on reasonable request.

6) PLOS ONE now requires that authors provide the original uncropped and unadjusted images underlying all blot or gel results reported in a submission’s figures or Supporting Information files. This policy and the journal’s other requirements for blot/gel reporting and figure preparation are described in detail at https://journals.plos.org/plosone/s/figures#loc-blot-and-gel-reporting-requirements and https://journals.plos.org/plosone/s/figures#loc-preparing-figures-from-image-files. When you submit your revised manuscript, please ensure that your figures adhere fully to these guidelines and provide the original underlying images for all blot or gel data reported in your submission. See the following link for instructions on providing the original image data: https://journals.plos.org/plosone/s/figures#loc-original-images-for-blots-and-gels.

In your cover letter, please note whether your blot/gel image data are in Supporting Information or posted at a public data repository, provide the repository URL if relevant, and provide specific details as to which raw blot/gel images, if any, are not available. Email us at plosone@plos.org if you have any questions.

**Reply**: Thanks for your comments. The original blot figure is supplied as supplement figure 1.

7) Please provide additional information about each of the cell lines used in this work, including any quality control testing procedures (authentication, characterisation, and mycoplasma testing). For more information, please see http://journals.plos.org/plosone/s/submission-guidelines#loc-cell-lines.

**Reply**: Thanks for your comments. As mentioned in Line 135-137, HA-VSMC were supplied by Shanghai Fusheng Industrial Co., Ltd. And HUCBSC was supplied by Qilu Cell Bank. The following figures are verified HUCBSC activity (Fig 2A).

8) At this time, we request that you please report additional details in your Methods section regarding animal care, as per our editorial guidelines:

(1) Please state the source of the rabbits used in the study (where they were purchased)

(2) Please include the method of euthanasia

(3) Please describe the post-operative care received by the animals, including the frequency of monitoring and the criteria used to assess animal health and well-being.

**Reply**: Thanks for your comments.

(1) The source of the rabbits was stated in the Methods section. (Page 5, Line 128-129)
(2) The method of euthanasia has been added. (Page 6, Line 173-174)

The post-operative care are as follows: After 15-20 minutes of compression and hemostasis after intervention, the bandage was pressurized and bandaged for fixation. After 4-6 hours, the bandage was removed. The blood glucose and body temperature of the experimental rabbits were monitored twice a day, morning and evening. Whether the puncture point was bleeding, infection, and the color change of the lower limbs during the operation were observed. The eating, drinking and activities of the experimental rabbits were also observed. In this experiment, one rabbit developed a high body temperature greater than 49 ℃, which was considered to be related to the skin infection at the surgical puncture point, and recovered after 3 days of anti-inflammatory administration of penicillin. Two rabbits suffered from lack of energy, which was considered to be hyperglycemia, and recovered after subcutaneous injection of small dose of insulin.（Page 6-7, Line 163-174)
